# Supplementary material for: De novo synthesis of a sunscreen compound in vertebrates
Source: eLife. 2015 May 12;4:e05919. doi: 10.7554/eLife.05919 (PMC4426668; doi:10.7554/eLife.05919)
Supplement: Supplementary file 1. — SPCs used for multiple sequence alignment and phylogenetic tree construction. DOI: http://dx.doi.org/10.7554/eLife.05919.021 [file elife05919s002.docx]

**Supplementary File 1.** Sugar Phosphate Cyclases (SPCs) used for multiple sequence alignment and phylogenetic tree construction.

| Family | Gene/Protein Name | Accession No. | Organism |
| --- | --- | --- | --- |
| **Bacterial EEVS** | BAD07382.1 | BAD07382.1 | *Actinoplanes* sp. A40644 |
|  | CetA | EF120454.2 | *Actinomyces* sp. Lu 9419 |
|  | AcbC | AEV84575.1 | *Actinoplanes* sp*.* SE50/110 |
|  | EEVS | WP_005152974.1 | *Amycolatopsis azurea* DSM 43854 |
|  | H074_RS00130 | WP_007027974.1 | *Amycolatopsis decaplanina* |
|  | H074_21207 | EME57750.1 | *Amycolatopsis decaplanina* DSM 44594 |
|  | EEVS | WP_020673085 | *Amycolatopsis nigrescens* |
|  | AroB | AGM08030.1 | *Amycolatopsis orientalis* HCCS10007 |
|  | EEVS | WP_006999601.1 | *Candidatus Burkholderia kirkii* |
|  | EEVS | CCD36718 | *Candidatus Burkholderia kirkii* UZHbot1 |
|  | Cja_3250 | ACE84801.1 | *Cellvibrio japonicus* Ueda107 |
|  | CLD_3207 | ACA45465.1 | *Clostridium botulinum* B1 str*. Okra* |
|  | Cpap_0968 | EGD46588.1 | *Clostridium papyrosolvens* DSM 2782 |
|  | D187_002969 | EPX59479.1 | *Cystobacter fuscus* DSM 2262 |
|  | AcbC | CBL44970.1 | *gamma proteobacterium* HdN1 |
|  | MESS4_430082 | CCV12436.1 | *Mesorhizobium* sp. STM 4661 |
|  | EEVS | WP_020731587.1 | *Mycobacterium marinum* |
|  | AroB_1 | ACC39042.1 | *Mycobacterium marinum* M |
|  | EEVS | EPQ71344.1 | *Mycobacterium marinum* MB2 |
|  | MMEU_4200 | EPQ72818.1 | *Mycobacterium marinum* str. *Europe* |
|  | EEVS | GAD87067.1 | *Nocardia asteroides* |
|  | NS07 CONTIG 00143-0015 | GAF31941.1 | *Nocardia seriolae* N-2927 |
|  | PrlA | ABL74380.1 | *Nonomuraea spiralis* |
|  | EEVS | ERV42559.1 | *Pseudomonas aeruginosa* |
|  | PflA506_4591 | AFJ55097.1 | *Pseudomonas fluorescens* A506 |
|  | EEVS | EPJ83049.1 | *Pseudomonas sp.* CFT9 |
|  | UUC_15323 | EIL99898.1 | *Rhodanobacter denitrificans* |
|  | EEVS | WP_008438647.1 | *Rhodanobacter thiooxydans* |
|  | UUA_15933 | EIL97123.1 | *Rhodanobacter thiooxydans* LCS2 |
|  | EEVS | WP_020113256.1 | *Rhodococcus* 114MFTsu3.1 |
|  | EEVS | WP_019667777.1 | *Rhodococcus* 29MFTsu3.1 |
|  | EEVS | WP_021331771 | *Rhodococcus erythropolis* |
|  | O5Y_25890 | AGT94995.1 | *Rhodococcus erythropolis* CCM2595 |
|  | N601_00990 | EQM35423.1 | *Rhodococcus erythropolis* DN1 |
|  | RER_54360 | BAH36144.1 | *Rhodococcus erythropolis* PR4 |
|  | EEVS | WP_021345782 | *Rhodococcus* sp. P27 |
|  | EEVS | WP_015102640 | *Saccharothrix espanaensis* DSM 44229 |
|  | Staur_1386 | ADO69190.1 | *Stigmatella aurantiaca* DW4/3-1 |
|  | EEVS | WP_010359798.1 | *Streptomyces acidiscabies* 84-104 |
|  | SalQ | ABV57470.1 | *Streptomyces albus* |
|  | SU9_09459 | EJJ07289.1 | *Streptomyces auratus* AGR0001 |
|  | EEVS | WP_005477027.1 | *Streptomyces bottropensis* ATCC 25435 |
|  | EEVS | WP_040902607.1 | *Streptomyces chartreusis* |
|  | SSCG_00526 | EDY47498.1 | *Streptomyces clavuligerus* ATCC 27064 |
|  | SMCF_997 | EHN79464.1 | *Streptomyces coelicoflavus* ZG0656 |
|  | GacC | CAL64849.1 | *Streptomyces glaucescens* GLA.O |
|  | VldA | ABC67267.1 | *Streptomyces hygroscopicus* subsp. *limoneus* |
|  | EEVS | AAZ91667.1 | *Streptomyces hygroscopicus* subsp*. yingchengensis* |
|  | EEVS | WP_009076280.1 | *Streptomyces* sp. AA4 |
|  | EEVS | WP_037784577.1 | *Streptomyces* sp. CNY228 |
|  | EEVS | AGZ94062.1 | *Streptomyces* sp. MMG1533 |
|  | EEVS | WP_026048441.1 | *Streptomyces* sp. S4 |
|  | EEVS | WP_007385523.1 | *Streptomyces sviceus* |
|  | SSEG_08792 | EDY55324.2 | *Streptomyces sviceus* ATCC 29083 |
|  | AciPR4_1231 | ADV82056 | *Terriglobus saanensis* SP1PR4 |
| **Animal EEVS** | LOC101799904 | XP_005011275.1 | *Anas platyrhynchos* |
|  | LOC100554413 | XP_003217873.2 | *Anolis carolinensis* |
|  | LOC103021483 | XP_007241787.1 | *Astyanax mexicanus* |
|  | UY3_08628 | EMP34204.1 | *Chelonia mydas* |
|  | LOC101935311 | XP_005282175.1 | *Chrysemys picta bellii* |
|  | A306_01079 | EMC89871.1 | *Columba livia* |
|  | LOC100003999 | XP_001343422.1 | *Danio rerio* |
|  | DLA_It04010 | CBN80976.1 | *Dicentrarchus labrax* |
|  | LOC102050204 | XP_005432702.1 | *Falco cherrug* |
|  | LOC101920037 | XP_005230087.1 | *Falco peregrinus* |
|  | LOC101811082 | XP_005053423.1 | *Ficedula albicollis* |
|  | ENSGMOG00000007414.1 | ENSGMOG00000007414 | *Gadus morhua* |
|  | LOC427594 | XP_425167.2 | *Gallus gallus* |
|  | ENSGACG00000011871 | ENSGACP00000015700 | *Gasterosteus aculeatus* |
|  | LOC102035384 | XP_005420282.1 | *Geospiza fortis* |
|  | LOC102309185 | XP_005947633.1 | *Haplochromis burtoni* |
|  | LOC102684922 | XP_006630707.1 | *Lepisosteus oculatus* |
|  | LOC101474077 | XP_004567457.1 | *Maylandia zebra* |
|  | LOC100539368 | XP_003210235.1 | *Meleagris gallopavo* |
|  | LOC101868264 | XP_005149534.1 | *Melopsittacus undulatus* |
|  | LOC102782305 | XP_006784803.1 | *Neolamprologus brichardi* |
|  | GSONMT00065608001 | CDQ61676.1 | *Oncorhynchus mykiss* |
|  | LOC100690451 | XP_003442831.1 | *Oreochromis niloticus* |
|  | LOC101163482 | XP_004068647.1 | *Oryzias latipes* |
|  | LOC102457108 | XP_006120116.1 | *Pelodiscus sinensis* |
|  | LOC103129387 | XP_007540516.1 | *Poecilia formosa* |
|  | LOC102106679 | XP_005522289.1 | *Pseudopodoces humilis* |
|  | LOC102205679 | XP_005726665.1 | *Pundamilia nyererei* |
|  | LOC100223651 | XP_002188776.1 | *Taeniopygia guttata* |
|  | LOC100492806 | XP_002940521.1 | *Xenopus (Silurana) tropicalis* |
|  | LOC102222998 | XP_005815791.1 | *Xiphophorus maculatus* |
| **Stramenopile EEVS** | AURANDRAFT_3740 | XP_009039347.1 | *Aureococcus anophagefferens* |
|  | CHC_T00009338001 | XP_005713525 | *Chondrus crispus* |
|  | COCSUDRAFT_39261 | XP_005652201.1 | *Coccomyxa subellipsoidea C-169* |
|  | CYME_CMP183C | XP_005537849 | *Cyanidioschyzon merolae* strain 10D |
|  | Esi_0086_0074 | CBJ27882 | *Ectocarpus siliculosus* |
|  | THAOC_37874 | EJK43661 | *Thalassiosira oceanica* |
|  | PHATRDRAFT_8772 | XP_002177202 | *Phaeodactylum tricornutum* |
|  | THAOC_37874 | EJK43661 | *Thalassiosira oceanica* |
|  | HAPSDRAFT_21539 | XP_002287560 | *Thalassiosira pseudonana* |
|  | CHC_T00009338001 | XP_005713525 | *Chondrus crispus* |
|  | Gasu_30570 | XP_005706140 | *Galdieria sulphuraria* |
| **EVS** | Amir_2000 | ACU35948.1 | *Actinosynnema mirum* DSM 43827 |
|  | Staur_3140 | ADO70932.1 | *Stigmatella aurantiaca* DW4/3-1 |
|  | DHQS | WP_002620792.1 | *Cystobacter fuscus* |
|  | DHQS | WP_02806414.1 | *Solirubrobacter soli* |
|  | DHQS | WP_015800837.1 | *Actinosynnema mirum* |
|  | DHQS | BAL88435.1 | *Actinoplanes missouriensis* |
|  | DHQS | WP_019435820 | *Streptomyces* sp. AA0539 |
|  | KF386858.1 | AGZ15443 | *Streptomyces* sp. MK498-98F14 |
|  | DHQS | WP_02550010 | *Streptomyces scabrisporus* |
| **Archaeal DHQS** | Ahos_1215 | WP_040876425.1 | *Acidianus hospitalis* |
|  | Calag_0109 | WP_013776014.1 | *Caldisphaera lagunensis* |
|  | Cmaq_0806 | AFZ69897.1 | *Caldivirga maquilingensis* |
|  | CM19_06260 | ABW01641.1 | *Candidatus acidianus copahuensis* |
|  | Igni_0018 | EZQ06961.1 | *Ignicoccus hospitalis* |
|  | Igag_1733 | ABU81202.1 | *Ignisphaera aggregans* |
|  | CP002656.1 | YP_003860410.1 | *Metallosphaera cuprina* |
|  | HA72_1062 | AEB94516.1 | *Metallosphaera sedula* |
|  | MetMK1DRAFT_00032710 | AIM27213.1 | *Metallosphaera yellowstonensis* |
|  | Pars_2111 | WP_009075654.1 | *Pyrobaculum arsenaticum* |
|  | Pcal_0895 | ABP51657.1 | *Pyrobaculum calidifontis* |
|  | AroB | YP_001055787.1 | *Sulfolobales archaeon* AZ1 |
|  | AroB | EWG07805.1 | *Sulfolobus islandicus* |
|  | AroB | YP_002829840.1 | *Sulfolobus solfataricus* |
|  | AroB | AAK40642.1 | *Sulfolobus tokodaii* |
|  | AroB | WP_010980356.1 | *Thermoproteus tenax* |
|  | Vdis_0216 | CCC82373.1 | *Vulcanisaeta distributa* |
|  | VMUT_1430 | ADN49628.1 | *Vulcanisaeta moutnovskia* |
| **Bacterial and fungal DHQS** | Amir_5253** | ACU39074.1 | *Actinosynnema mirum* DSM 43827 |
|  | Ava_4386 | ABA23984.1 | *Anabaena variabilis* ATCC 29413 |
|  | An1DQS | XP_658312.1 | *Aspergillus nidulans* |
|  | BsDHQS | AAA20860.1 | *Bacillus subtilis* |
|  | DHQS | CDH47441 | *Candidatus Contendobacter odensis* |
|  | EcDHQS | AAA58186.1 | *Escherichia coli* str. K-12 |
|  | C695_RS01450 | WP_001156090.1 | *Helicobacter pylori* |
|  | DHQS | WP_020681978 | *Marinobacterium rhizophilum* |
|  | DHQS | WP_009725480 | *Methylophaga lonarensis* |
|  | DHQS | WP_008290485 | *Methylophaga thiooxydans* |
|  | Npun_5729 | ACC84029.1 | *Nostoc punctiforme* PCC 73102 (ATCC 29133) |
|  | DHQS | WP_023970131 | *Pseudomonas chlororaphis* |
|  | DHQS | WP_015479237 | *Pseudomonas denitrificans* |
|  | PKB_5345 | CDF86657 | *Pseudomonas knackmussii* B13 |
|  | AU05_25215 | EZH77367 | *Pseudomonas pseudoalcaligenes* AD6 |
|  | Sa1XAG | 1XAG_A | *Staphylococcus aureus* |
|  | Staur_4041** | ADO71827.1 | *Stigmatella aurantiaca* DW4/3-1 |
|  | P354_02295 | EXU86293 | *Streptomyces albulus* |
|  | DHQS | WP_0066074643 | *Streptomyces auratus* |
|  | DHQS | WP_004942390 | *Streptomyces mobaraensis* |
|  | DHQS | WP_005319844 | *Streptomyces pristinaespiralis* ATCC_25486 |
|  | DHQS | WP_019884829 | *Streptomyces purpureus* |
|  | DHQS | WP_003984693 | *Streptomyces rimosus* |
|  | DHQS | WP_026249565 | *Streptomyces* sp. ATexAB-D23 |
|  | DHQS | WP_026359219 | *Streptomyces* sp. DvalAA-83 |
|  | DHQS | WP_016467710 | *Streptomyces* sp. HPH0547 |
|  | DHQS | WP_018087611 | *Streptomyces* sp. FxanaC1 |
|  | DHQS | WP_018539828 | *Streptomyces* sp. MspMP-M5 |
|  | TTHA1386 | YP_144652.1 | *Thermus thermophilus* HB8 |
|  | DHQS | WP_012639562 | *Thioalkalivibrio sulfidophilus* |
|  | DHQS | WP_026186219 | *Thioalkalivibrio thiocyanodenitrificans* |
| **Plant and algal DHQS** | AT5G66120 | NP_56029 | *Arabidopsis thaliana* |
|  | LOC100834750 | XP_003578532 | *Brachypodium distachyon* |
|  | CARUB_v10026413mg | XP_006280477 | *Capsella rubella* |
|  | LOC102631297 | XP_006466989.1 | *Citrus sinensis* |
|  | COCSUDRAFT_35806 | XP_005649993 | *Coccomyxa subellipsoidea* C-169 |
|  | EUTSA_v10004219mg | XP_00639797 | *Eutrema salsugineum* |
|  | LOC102714768 | XP_006661484 | *Oryza brachyantha* |
|  | Os09g0539100 | NP_001063802 | *Oryza sativa japonica* |
|  | EF678425.1 | ABR18182 | *Picea sitchensis* |
|  | LOC101782627 | XP_004957492 | *Setaria italica* |
|  | LOC102598775 | XP_006340763 | *Solanum tuberosum* |
|  | BT043106.1 | ACF88111 | *Zea mays* |
|  | PDE_00008 | WP_018334610.1 | *Actinomycetospora chiangmaiensis* |
| **DDGS** | Amir_4259 | ACU38114.1 | *Actinosynnema mirum* DSM 43827 |
|  | Ava_3858 | ABA23463.1 | *Anabaena variabilis* ATCC 29413 |
|  | DDGS | BAO51913.1 | *Aphanothece halophytica* |
|  | ACLA_055850 | EAW13537.1 | *Aspergillus clavatus* NRRL 1 |
|  | BAUCODRAFT_80557 | EMC91075.1 | *Baudoinia compniacensis* UAMH 10762 |
|  | BBA_00472 | EJP70842.1 | *Beauveria bassiana* ARSEF 2860 |
|  | COCC4DRAFT_167163 | ENI05767.1 | *Bipolaris maydis* ATCC 48331 |
|  | COCHEDRAFT_1194844 | EMD91152.1 | *Bipolaris maydis* C5 |
|  | COCMIDRAFT_8170 | EUC42205.1 | *Bipolaris oryzae* ATCC 44560 |
|  | COCSADRAFT_38955 | EMD62170.1 | *Bipolaris sorokiniana* ND90Pr |
|  | BcDW1_9470 | EMR81915.1 | *Botryotinia fuckeliana* BcDW1 |
|  | BofuT4_P133930.1 | CCD53839.1 | *Botryotinia fuckeliana* T4 |
|  | DDGS | AFZ02505 | *Calothrix* sp. PCC 6303 |
|  | DDGS | WP_019490229.1 | *Calothrix* sp. PCC 7103 |
|  | A1O1_01840 | EXJ93448.1 | *Capronia coronata* CBS 617.96 |
|  | Cha6605_2820 | AFY93856.1 | *Chamaesiphon minutus* PCC 6605 |
|  | DDGS | WP_016876765.1 | *Chlorogloeopsis fritschii* |
|  | Chro_0778 | AFY86324.1 | *Chroococcidiopsis thermalis* PCC 7203 |
|  | G647_03988 | ETI24619.1 | *Cladophialophora carrionii* CBS 160.54 |
|  | A1O5_01012 | EXJ76504.1 | *Cladophialophora psammophila* CBS 110553 |
|  | A1O7_04691 | EXJ60538.1 | *Cladophialophora yegresii* CBS 114405 |
|  | CPUR_02718 | CCE29027.1 | *Claviceps purpurea* 20.1 |
|  | CFIO01_11686 | EXF78170.1 | *Colletotrichum fioriniae* PJ7 |
|  | CGLO_11575 | EQB49116.1 | *Colletotrichum gloeosporioides* Cg-14 |
|  | CGGC5_4437 | XP_007274966.1 | *Colletotrichum gloeosporioides* Nara gc5 |
|  | GLRG_05915 | EFQ30771.1 | *Colletotrichum graminicola* M1.001 |
|  | Cob_10738 | ENH80676.1 | *Colletotrichum orbiculare* MAFF 240422 |
|  | W97_04284 | EON65049.1 | *Coniosporium apollinis* CBS 100218 |
|  | CCM_06613 | EGX90194.1 | *Cordyceps militaris* CM01 |
|  | Cri9333_2379 | AFZ13246.1 | *Crinalium epipsammum* PCC 9333 |
|  | Cylst_1339 | AFZ23628.1 | *Cylindrospermum stagnale* PCC 7417 |
|  | HMPREF1541_10826 | ETN43961.1 | *Cyphellophora europaea* CBS 101466 |
|  | DACRYDRAFT_108509 | EJU01177.1 | *Dacryopinax* sp. DJM-731 SS1 |
|  | DDGS | WP_015229181 | *Dactylococcopsis salina* |
|  | DOTSEDRAFT_74971 | EME40344.1 | *Dothistroma septosporum* NZE10 |
|  | EPUS_06787 | ERF68371.1 | *Endocarpon pusillum* Z07020 |
|  | HMPREF1120_03313 | EHY55163.1 | *Exophiala dermatitidis* NIH/UT8656 |
|  | DDGS | WP_016867391.1 | *Fischerella muscicola* |
|  | FFUJ_02302 | CCT65366.1 | *Fusarium fujikuroi* IMI 58289 |
|  | FGSG_07578.1 | ESU13851.1 | *Fusarium graminearum* PH-1 |
|  | FOPG_14554 | EXL69517.1 | *Fusarium oxysporum* f. sp. *conglutinans* race 2 54008 |
|  | FOC1_g10007978 | ENH63840.1 | *Fusarium oxysporum* f. sp. *cubense* race 1 |
|  | FOC4_g10004309 | EMT72824.1 | *Fusarium oxysporum* f. sp*. cubense* race 4 |
|  | FOWG_01820 | EWZ97333.1 | *Fusarium oxysporum* f. sp*. lycopersici* MN25 |
|  | FOMG_05909 | EXK43277.1 | *Fusarium oxysporum* f. sp*. melonis* 26406 |
|  | FOVG_03599 | EXA51127.1 | *Fusarium oxysporum* f. sp*. pisi* HDV247 |
|  | FOCG_01565 | EXL63199.1 | *Fusarium oxysporum* f. sp. *radicis-lycopersici* 26381 |
|  | FOQG_12197 | EXK83496.1 | *Fusarium oxysporum* f. sp*. raphani* 54005 |
|  | FOTG_14331 | EXM17492.1 | *Fusarium oxysporum* f. sp. *vasinfectum* 25433 |
|  | FOZG_06058 | EWZ45846.1 | *Fusarium oxysporum* Fo47 |
|  | FOXB_11899 | EGU77611.1 | *Fusarium oxysporum* Fo5176 |
|  | FOYG_03768 | EWY99830.1 | *Fusarium oxysporum* FOSC 3-a |
|  | FPSE_08031 | EKJ71763.1 | *Fusarium pseudograminearum* CS3096 |
|  | FVEG_12691 | EWG54478.1 | *Fusarium verticillioides* 7600 |
|  | GLAREA_08216 | EPE24364.1 | *Glarea lozoyensis* ATCC 20868 |
|  | GLOTRDRAFT_39501 | XP_007864776.1 | *Gloeophyllum trabeum* ATCC 11539 |
|  | DDGS | WP_023072000 | *Leptolyngbya* sp. Heron Island J |
|  | DDGS | WP_006516570 | *Leptolyngbya* sp. PCC 7375 |
|  | LEMA_P063060.1 | CBX90180.1 | *Leptosphaeria maculans* JN3 |
|  | DDGS | WP_023068561.1 | *Lyngbya aestuarii* |
|  | L8106_16364 | EAW37588.1 | *Lyngbya* sp. PCC 8106 |
|  | MPH_07850 | EKG14950.1 | *Macrophomina phaseolina* MS6 |
|  | MGG_00016 | EHA49547.1 | *Magnaporthe oryzae* 70-15 |
|  | OOU_Y34scaffold01060g1 | ELQ32736.1 | *Magnaporthe oryzae* Y34 |
|  | MBM_04236 | EKD17375.1 | *Marssonina brunnea* f. sp. *multigermtubi* MB_m1 |
|  | MELLADRAFT_46120 | XP_007418557.1 | *Melampsora larici-populina* 98AG31 |
|  | MAC_00588 | EFY93350.1 | *Metarhizium acridum* CQMa 102 |
|  | FVEG_12691 | WP_017655453.1 | *Microchaete* sp. PCC 7126 |
|  | DDGS | WP_002794106.1 | *Microcystis aeruginosa* |
|  | C789_465 | ELS49746.1 | *Microcystis aeruginosa* DIANCHI905 |
|  | IPF_3031 | CAO90104.1 | *Microcystis aeruginosa* PCC 7806 |
|  | AcbC | CCI02410.1 | *Microcystis aeruginosa* PCC 9443 |
|  | AcbC | CCH99802.1 | *Microcystis aeruginosa* PCC 9717 |
|  | AcbC | CCI19960.1 | *Microcystis aeruginosa* PCC 9807 |
|  | MICAG_2780005 | CCI25385.1 | *Microcystis aeruginosa* PCC 9808 |
|  | E5Q_03910 | GAA97234.1 | *Mixia osmundae* IAM 14324 |
|  | DDGS | WP_014813469.1 | *Mycobacterium chubuense* |
|  | DDGS | AFM14977.1 | *Mycobacterium chubuense* NBB4 |
|  | NECHADRAFT_48307 | XP_003043726.1 | *Nectria haematococca* mpVI 77-13-4 |
|  | UCRNP2_5834 | EOD47414.1 | *Neofusicoccum parvum* UCRNP2 |
|  | N9414_08103 | EAW44170.1 | *Nodularia spumigena* CCY9414 |
|  | Npun_R5600 | ACC83905.1 | *Nostoc punctiforme* PCC 73102 |
|  | Nos7524_3370 | AFY49165.1 | *Nostoc* sp. PCC 7524 |
|  | OCS_06803 | EQK97484.1 | *Ophiocordyceps sinensis* CO18 |
|  | Osc7112_3782 | AFZ08125.1 | *Oscillatoria nigro-viridis* PCC 7112 |
|  | PDE_00008 | EPS25077.1 | *Penicillium oxalicum* 114-2 |
|  | PFICI_12759 | ETS75815.1 | *Pestalotiopsis fici* W106-1 |
|  | MYCFIDRAFT_33875 | XP_007931255.1 | *Pseudocercospora fijiensis* CIRAD86 |
|  | DDGS | WP_010243321.1 | *Pseudonocardia* sp. P1 |
|  | PaG_02576 | ETS62823 | *Pseudozyma aphidis* DSM 70725 |
|  | PFL1_03740 | EPQ28940.1 | *Pseudozyma flocculosa* PF-1 |
|  | PTT_06860 | EFQ95201.1 | *Pyrenophora teres* f. *teres* 0-1 |
|  | PTRG_02787 | EDU45310.1 | *Pyrenophora tritici-repentis* Pt-1C-BFP |
|  | PCON_03344 | CCX16645 | *Pyronema omphalodes* CBS 100304 |
|  | DDGS | WP_020111281.1 | *Rhodococcus* sp. 114MFTsu3.1 |
|  | DDGS | WP_019663384.1 | *Rhodococcus* sp. 29MFTsu3.1 |
|  | DDGS | WP_022606420 | *Rubidibacter lacunae* |
|  | SBOR_4234 | ESZ95378.1 | *Sclerotinia borealis* F-4157 |
|  | SS1G_08336 | EDN92473.1 | *Sclerotinia sclerotiorum* 1980 UF-70 |
|  | DDGS | WP_017743132.1 | *Scytonema hofmanni* |
|  | SETTUDRAFT_100700 | EOA81028.1 | *Setosphaeria turcica* Et28A |
|  | SEPMUDRAFT_151827 | EMF08929.1 | *Sphaerulina musiva* SO2202 |
|  | sr12669 | CBQ71813.1 | *Sporisorium reilianum* SRZ2 |
|  | STEHIDRAFT_146260 | EIM88185.1 | *Stereum hirsutum* FP-91666 SS1 |
|  | UCRPA7_3232 | EOO01292.1 | *Togninia minima* UCRPA7 |
|  | UHOR_02376 | CCF53523.1 | *Ustilago hordei* |
|  | VDBG_08620 | EEY22510.1 | *Verticillium alfalfae* VaMs.102 |
|  | VDAG_08289 | EGY17125.1 | *Verticillium dahliae* VdLs.17 |
|  | DDGS | WP_006509782 | *Xenococcus* sp. PCC 7305 |
|  | MYCGRDRAFT_76728 | XP_003848682.1 | *Zymoseptoria tritici* IPO323 |
| **DHQS-like** | Npun_5231* | ACC83559.1 | *Nostoc punctiforme* PCC 73102 (ATCC 29133) |
|  | Npun_1267* | ACC79988.1 | *Nostoc punctiforme* PCC 73102 (ATCC 29133) |
| **aDHQS** | Amir_3296*** | ACU37202.1 | *Actinosynnema mirum* DSM 43827 |
|  | Asm47 | AAC14006.1 | *Actinosynnema pretiosum* subsp*. auranticum* |
|  | GdmO | AAO06928.1 | *Streptomyces hygroscopicus* |
|  | MitP | AAD28456.1 | *Streptomyces lavendulae* |
|  | RifG | AAC01717.1 | *Amycolatopsis mediterranei* S699 |
| **DOIS** | KanA | BAD20759.1 | *Streptomyces kanamyceticus* |
|  | RbmA | CAG34037.1 | *Streptomyces ribosidificus* |
|  | NemA | BAD95820.1 | *Streptomyces fradiae* |
|  | GntB | AAR98548.1 | *Micromonospora echinospora* |
|  | BtrC | BAA83344.1 | *Bacillus circulans* |
|  | TbmA | CAE22471.1 | *Streptoalloteichus tenebrarius* |

*Predicted DHQS-like proteins found in the *N. punctiforme* genome*.* **Predicted DHQS-like proteins found in the *A. mirum* and *S. aurantiaca* genomes. ***Predicted aDHQS protein found in the *A. mirum* genome*.* Abbreviations: EEVS; 2-epi-5-epi-valiolone synthase, DDGS; 4-desmethyl-4-deoxygadusol synthase, EVS; 2-epi-valiolone synthase, DHQS; 3-dehydroquinate synthase, aDHQS; aminoDHQ synthase, DOIS; 2-deoxy-*scyllo*-inosose synthase.
