# Supplementary material for: De novo synthesis of a sunscreen compound in vertebrates
Source: eLife. 2015 May 12;4:e05919. doi: 10.7554/eLife.05919 (PMC4426668; doi:10.7554/eLife.05919)
Supplement: Supplementary file 2. — MT-Ox proteins used for multiple sequence alignment and phylogenetic tree construction. DOI: http://dx.doi.org/10.7554/eLife.05919.022 [file elife05919s003.docx]

**Supplementary File 2.** MT-Ox proteins used for multiple sequence alignment and phylogenetic tree construction.

| **Family** | **Gene symbol** | **Accession No.** | **Organism** |
| --- | --- | --- | --- |
|  | LOC101799721 | XP_005011274 | *Anas platyrhynchos* |
|  | LOC100554218 | XP_008103594 | *Anolis carolinensis* |
|  | LOC103021811 | XP_007241788.1 2 | *Astyanax mexicanus* |
|  | LOC101935589 | XP_005282176.1 | *Chrysemys picta bellii* |
|  | LOC102090989 | XP_005514955.1 | *Columba livia* |
|  | zgc:113054 | NP_001013468.1 | *Danio rerio* |
|  | DLA_It04000 | CBN80975.1 | *Dicentrarchus labrax* |
|  | LOC102050380 | XP_005432703 | *Falco cherrug* |
|  | LOC101919857 | XP_005230086 | *Falco peregrinus* |
|  | LOC101811274 | XP_005053424 | *Ficedula albicollis* |
|  | ENSGMOG00000007404 | ENSGMOP00000007916 | *Gadus morhua* |
|  | LOC427595 | XP_425168.3 | *Gallus gallus* |
|  | ENSGACG00000011845 | ENSGACP00000015696 | *Gasterosteus aculeatus* |
|  | LOC102035220 | XP_005420281.1 | *Geospiza fortis* |
|  | LOC102308870 | XP_005943916 | *Haplochromis burtoni* |
|  | LOC102695979 | XP_006630675.1 | *Lepisosteus oculatus* |
|  | LOC101474366 | XP_004567458.1 | *Maylandia zebra* |
|  | LOC100539521 | XP_003210236 | *Meleagris gallopavo* |
|  | LOC101868426 | XP_005149535 | *Melopsittacus undulatus* |
|  | LOC102782600 | XP_006784804.1 | *Neolamprologus brichardi* |
|  | GSONMT00065609001 | CDQ61677.1 | *Oncorhynchus mykiss* |
|  | LOC100697673 | XP_005450406.1 | *Oreochromis niloticus* |
|  | LOC101163242 | XP_004068646.1 | *Oryzias latipes* |
|  | LOC102457357 | XP_006120117.1 | *Pelodiscus sinensis* |
|  | LOC103129385 | XP_007540514.1 | *Poecilia formosa* |
|  | LOC102106494 | XP_005522288 | *Pseudopodoces humilis* |
|  | LOC102205957 | XP_005726666.1 | *Pundamilia nyererei* |
|  | LOC100220728 | XP_002188799 | *Taeniopygia guttata* |
|  | MGC147226 | NP_001072630 | *Xenopus (Silurana) tropicalis* |
|  | LOC102222561 | XP_005814009.1 | *Xiphophorus maculatus* |
