# Supplementary material for: De novo synthesis of a sunscreen compound in vertebrates
Source: eLife. 2015 May 12;4:e05919. doi: 10.7554/eLife.05919 (PMC4426668; doi:10.7554/eLife.05919)
Supplement: Supplementary file 3. — Reciprocal best hit analysis. DOI: http://dx.doi.org/10.7554/eLife.05919.023 [file elife05919s004.docx]

**Supplementary File 3.** Reciprocal best hit analysis

| Unique bacterial EEVS subjects | Unique vertebrate EEVS subjects |
| --- | --- |
| *Amycolatopsis decaplanina* (WP_007027974.1) | *Anas platyrhynchos* (XP_005011275.1) |
| *Actinoplanes* sp*.* A40644 (BAD07382.1) | *Chrysemys picta bellii* (XP_005282175.1) |
| *Actinomyces* sp. Lu 9419 (CetA) | *Dicentrarchus labrax* (CBN80976.1) |
| *Cystobacter fuscus* DSM 2262 (EPX59479.1 | *Falco peregrinus* (XP_005230087.1) |
| *Nonomuraea spiralis* (PrlA) | *Gallus gallus* (XP_425167.2) |
| *Streptomyces acidiscabies* 84-104 (WP_010359798.1) | *Gasterosteus aculeatus* (ENSGACP00000015700) |
| *Streptomyces* sp. S4 (WP_026048441.1) | *Lepisosteus oculatus* (XP_006630707.1) |
|  | *Maylandia zebra* (XP_004567457.1) |
|  | *Meleagris gallopavo* (XP_003210235.1) |
|  | *Melopsittacus undulates* (XP_005149534.1) |
|  | *Oreochromis niloticus* (XP_003442831.1) |
|  | *Oryzias latipes* (XP_004068647.1) |
|  | *Pelodiscus sinensis* (XP_006120116.1) |
|  | *Xenopus (Silurana) tropicalis* (XP_002940521.1) |
|  | *Xiphophorus maculatus* (XP_005815791.1) |

A reciprocal blast hit analysis was performed on the EEVS proteins. First, the 55 bacterial EEVS sequences were blasted against the vertebrate EEVS sequences, which hit 17 unique vertebrate EEVS subjects. Then the vertebrate EEVS sequences were blasted against all proteins (minus vertebrate proteins) used for phylogenetic analysis, which hit 7 unique bacterial EEVS subjects. Two pairs of reciprocal best hits were identified; *Actinoplanes* sp. A40644 (BAD07382.1) was best hit with *Falco peregrinus* (XP_005230087.1), and *Cystobacter fuscus* DSM 2262 (EPX59479.1) with *Dicentrarchus labrax* (CBN80976.1).
