# Supplementary material for: De novo synthesis of a sunscreen compound in vertebrates
Source: eLife. 2015 May 12;4:e05919. doi: 10.7554/eLife.05919 (PMC4426668; doi:10.7554/eLife.05919)
Supplement: Supplementary file 4. — Yeast strains used. DOI: http://dx.doi.org/10.7554/eLife.05919.024 [file elife05919s005.docx]

**Supplementary File 4.** Yeast strains used

| **Strain** | **Genotype** | **Source** |
| --- | --- | --- |
| S288c | *MATα SUC gal mal mel flo1 flo8-1 hap bio1 bio6* | ATCC 204508, Manassas, VA |
| BY4742 *tal1∆* | *MATα his3∆1 leu2∆0 lys2∆0 ura3∆0 tal1∆::KanMX4* | Thermo Fisher Scientific Inc., Waltham, MA |
| BY4742 *tal1∆ trp1∆* | *MATα his3∆1 leu2∆0 lys2∆0 ura3∆0 tal1∆::KanMX4 trp1∆::URA3* | This study |
| BY4742 *tal1∆ trp1∆ rad1Δ* | *MATα his3∆1 leu2∆0 lys2∆0 ura3∆0 tal1∆::KanMX4 trp1∆::URA3 rad1Δ::LEU2* | This study |
| BY4742 *tal1∆ trp1∆/*pXP416 pXP420 | *MATα his3∆1 leu2∆0 lys2∆0 ura3∆0 tal1∆::KanMX4 trp1∆::URA3/* pXP416 pXP420 | This study |
| BY4742 *tal1∆ trp1∆/*pXP416-MTOX pXP420-EEVS | *MATα his3∆1 leu2∆0 lys2∆0 ura3∆0 tal1∆::KanMX4 trp1∆::URA3*/pXP416-EEVS pXP420-MTOX | This study |
| BY4742 *tal1∆ trp1∆ rad1Δ/* pXP416 pXP420 | *MATα his3∆1 leu2∆0 lys2∆0 ura3∆0 tal1∆::KanMX4 trp1∆::URA3 rad1Δ::LEU2/*pXP416 pXP420 | This study |
