# Supplementary material for: De novo synthesis of a sunscreen compound in vertebrates
Source: eLife. 2015 May 12;4:e05919. doi: 10.7554/eLife.05919 (PMC4426668; doi:10.7554/eLife.05919)
Supplement: Supplementary file 5. — Plasmids used. DOI: http://dx.doi.org/10.7554/eLife.05919.025 [file elife05919s006.docx]

**Supplementary File 5.** Plasmids used

| **Plasmid** | **Insert** | **Source/reference** |
| --- | --- | --- |
| pUC57-EEVS | EEVS (*Eco*RV*)* | GeneScript USA Inc. |
| pUC57-MTOX | MT-Ox (*Eco*RV*)* | GeneScript USA Inc. |
| pRSETB-EEVS | EEVS (*Bgl*II) | This study |
| pRSETB-MTOX | MT-Ox (*Bgl*II) | This study |
| pXP416 | none | Fang et al. 2011 (***Fang et al., 2011***); Addgene, Cambridge, MA |
| pXP416-MTOX | MT-OX (*Spe*I/*Xho*I) | This study |
| pXP20 | none | Fang et al. 2011 (***Fang et al., 2011***); Addgene, Cambridge, MA |
| pXP420-EEVS | EEVS (*Spe*I/*Xho*I) | This study |

Fang F, Salmon K, Shen MW, Aeling KA, Ito E, Irwin B, Tran UP, Hatfield GW, Da Silva NA, Sandmeyer S. 2011. A vector set for systematic metabolic engineering in *Saccharomyces cerevisiae*. *Yeast* **28**:123-136 doi:10.1002/yea.1824
