# Supplementary material for: De novo synthesis of a sunscreen compound in vertebrates
Source: eLife. 2015 May 12;4:e05919. doi: 10.7554/eLife.05919 (PMC4426668; doi:10.7554/eLife.05919)
Supplement: Supplementary file 6. — Primers used. DOI: http://dx.doi.org/10.7554/eLife.05919.026 [file elife05919s007.docx]

**Supplementary File 6.** Primers used

| **Primer** | **Sequence (5´→3´)^a^** | **Purpose** |
| --- | --- | --- |
| TRP1DisURA3UP | TATAGGAAGCATTTAATAGAACAGCATCGTAATATATGTGTACTTTGCAGTTATGACGCCGAAATTGAGGCTACTGCGCC | *TRP1* deletion |
| TRP1DisURA3LO | CCTGTGAACATTCTCTTCAACAAGTTTGATTCCATTGCGGTGAAATGGTAAAAGTCAACCGGCAGCGTTTTGTTCTTGGA | *TRP1* deletion |
| RAD1DisLEU2UP | GAGCATTTGCTAAATGTGTAAAAATAATATTGCACTATC CTGTTGAAAATATCTTTCCAGCACTGTTCACGTCGCACCTA | *RAD1* deletion |
| RAD1DisLEU2LO | CTATAGTTAATCGCATTTTATACTGATGTTTTAACAGGGTTCGTTAAATTAAACAATATTGCTGCATTAATGAATCGGCCA | *RAD1* deletion |
| TRP1DisUP | CTCACCCGCACGGCAGAGAC | Confirmation |
| TRP1DisLO | TGCCGGCGGTTGTTTGCAAG | Confirmation |
| URA3DisUp | GTGGCTGTGGTTTCAGGGTCCA | Confirmation |
| RAD1UP | CCTGAAGTGTTCTCTGTTTGCC | Confirmation |
| RAD1LO | GCTCAGATTCCACCAAATACGG | Confirmation |
| DEEVSUP | AGATCCACTAGTATGGAACGTCCGGGCGAAAC | EEVS cloning |
| DEEVSLO | TAGCCACTCGAGTCACTGCGGTGAGCCGGT | EEVS cloning |
| MTOXUP | AGATCCACTAGTATGCAAACGGCAAAAGTCTC | MTOX cloning |
| MTOXLO | TAGCCACTCGAGTCACCACAGAGACTGACCG | MTOX cloning |
| DEEVS-q-F | CCATCTGTTCACCGGGACAA | qPCR EEVS |
| DEEVS-q-R | TGCTGGGGTCAAGAAGGTTT | qPCR EEVS |
| MTOX-q-F | AGTAGAGCAGGTCATCATCCCT | qPCR MTOX |
| MTOX-q-R | CTATGATGGCGACTTTGGCTC | qPCR MTOX |

^a^*Spe*I and *Xho*I restriction sites are underlined
